# The tumor microenvironment in triple negative breast cancer and a strategy to improve responses to immunotherapy using cryoablation and immunostimulants

**DOI:** 10.1080/15384047.2026.2683942

**Published:** 2026-06-05

**Authors:** Ritvik Illindala, Yunpeng Yang, Tyler Mandt, Nicholas Webster, Nicole Steinmetz, Isabel Newton

**Affiliations:** a Department of Radiology, University of California San Diego, La Jolla, United States; b VA San Diego Healthcare System Research Service, La Jolla, United States; c Department of Medicine, Division of Endocrinology and Metabolism, University of California San Diego, La Jolla, United States; d Aiiso Yufeng Li Family Department of Chemical and Nano Engineering, University of California San Diego, La Jolla, United States; e VA San Diego Medical Center, Department of Interventional Radiology, La Jolla, United States

**Keywords:** Cryoablation, tumor microenvironment, breast cancer, triple-negative breast cancer, locoregional therapy, immunotherapy, immunostimulant, immune checkpoint inhibitor

## Abstract

One in eight women will develop breast cancer (BC) over their lifetime. Triple-negative breast cancer (TNBC) accounts for up to 20% of BC cases and has fewer treatment options, greater metastatic potential, a higher risk of recurrence, and a poorer prognosis compared to other BC subtypes. Compared to hormone receptor-positive BCs, TNBC has more tumor-infiltrating lymphocytes, a higher tumor mutational burden, and greater programmed death ligand-1 (PD-L1) expression. While these features suggest greater immunogenicity, the balance of the complex TNBC tumor microenvironment (TME) is immunosuppressive. The TME leads to modest and variable effects of immune checkpoint inhibitors (ICI) for TNBC. This review explores the dysfunctional immunological state of TNBC and proposes a multi-modal strategy integrating cryoablation as a source of tumor-associated antigens (TAAs) and immunostimulatory agents to reprogram antitumor immunity. It specifically explores the strategy of combining ICI and immunostimulants with cryoablation as a source of TAAs.

## Introduction

Breast cancer (BC) is one of the most challenging tumors to treat, despite significant advances in medical and surgical cancer therapy. Triple-negative breast cancer (TNBC), which lacks expression of estrogen receptor (ER), progesterone receptor (PR), and HER2, represents one of the most aggressive BC subtypes and is associated with unfavorable outcomes. While TNBC exhibits some features of cancers that respond well to immunotherapy, clinical responses to immune checkpoint inhibitors (ICI) have been modest, at best.

The TNBC tumor microenvironment (TME) portrays a complicated balance that overall favors immune suppression and enables tumor progression. While prior reviews have examined cryoablation and immunotherapy in breast cancer, this review specifically explores the unique immunological components of the TNBC TME and suggests a strategy to convert the TME from “cold” to “hot,” specifically by combining ICI to relieve T-cell exhaustion, with cryoablation, as a source of tumor-associated antigens (TAA), and immunostimulants to enhance TAA recognition.

## Materials and methods

A semi-systematic literature review was conducted to identify studies evaluating the TME of TNBC, cryoablation, immunotherapy, and immunostimulatory strategies. Searches were performed in PubMed, Google Scholar, and Embase for studies published between 2000 and 2026. Additional studies were identified through backward citation tracking of included articles. Relevant seminal papers published prior to 2000 were included.

Search terms included combinations of “triple-negative breast cancer”, “tumor microenvironment”, “cryoablation”, “immune checkpoint inhibitor”, “immunotherapy” “immunostimulants”, “CpG oligodeoxynucleotides”, “immunogenic cell death”, “immune checkpoints”, “immune cells”, “macrophages”, “T cells”, “epidemiology”, “clinical trials”, “preclinical studies”, and “limitations”. Additional articles were identified through manual review of reference lists from relevant articles.

Eligible studies included preclinical studies, clinical trials, cohort studies, meta-analyzes, and review articles relevant to TNBC or mechanistically translatable tumor models. Exclusion criteria included nonoriginal research without primary data, nonpeer-reviewed reports, studies lacking relevant immunological or clinical outcomes, non-TNBC populations without clear translational value, poor methodological quality, and non-English publications. All included studies were assessed in full-text form for relevance and methodological clarity.

## Breast cancer epidemiology and current trials

Breast cancer (BC) is the most common cancer worldwide and the second leading cause of cancer-related death among women in the United States. According to the American Cancer Society, one in eight women will develop invasive BC during their lifetime, and approximately 1/5th of them will die from their disease.^1^ Most BCs express ER and/or PR, and a subset of BCs overexpress HER2, with or without hormone receptor (HR) expression. Triple-negative breast cancer (TNBC) accounts for 15–20% of all BCs and more commonly affects young women, black women, and those with BRCA1 mutations.^2^ TNBC is more likely to present as metastatic disease and carries a poorer prognosis. Compared with HR+/HER2− BC, TNBC carries a threefold higher overall mortality risk and a sixfold higher risk of death within the first 2 y after diagnosis.^3^

Although advances in targeted therapies have improved outcomes for HR+ and HER2+ BCs, no such options exist for TNBC and outcomes continue to be poor. Chemotherapy remains the backbone of TNBC treatment, but the risk of recurrence is high, particularly within the first three to 5 y.^4^ Among patients diagnosed with BC, those with TNBC had higher rates of distant recurrence and four times the risk of visceral metastasis within 5 y.^4^

The response of TNBC to immune checkpoint inhibitors (ICIs) has been variable and more modest than for other cancers. In the KEYNOTE-355 phase III trial of chemotherapy with or without pembrolizumab (anti-PD-1 antibody), TNBC patients whose tumors had high PD-L1 expression had improved progression-free survival (PFS) of 9.7 months compared to 5.6 months with chemotherapy alone.^5^ Based on this modest improvement, the FDA thereby approved this combination for TNBC with high PD-L1 expression. Another phase III trial of TNBC, IMpassion130, added the anti-PD-L1 antibody atezolizumab to albumin-bound paclitaxel and improved overall survival (OS) from 18 to 25 months in previously untreated TNBC patients with locally advanced/metastatic disease.^6^ These modest improvements have not been observed consistently across trials. On follow-up trials, no survival benefit was seen with atezolizumab plus chemotherapy in both untreated and early relapsing disease (IMpassion 131) or just early relapsing disease (IMpassion 132).^7^ ^,^ ^8^ These trials had TNBC populations with similar PD-L1 expression which suggests that solely PD-L1 expression is not a sufficient predictor for response. Other regulatory mechanisms are present, which will be discussed later. By enhancing the immunogenicity of the TME and addressing the mechanisms of immune evasion, therapies like cryoablation combined with immunostimulants and ICIs offer potential to improve TNBC outcomes by stimulating systemic antitumor immunity.

## Immune state in TNBC

The weaker responses of TNBC to immunotherapy reflect a complex immune landscape governed by the TME. Whether antitumoral immunity is induced rather than suppressed relies on many aspects of the TME. These include the availability of TAA, effective initiation and maintenance of TAA-specific T-cell responses, a tumor mutational burden (TMB) sufficient to attract immune recognition, immune cell polarization, and the prevalence of immune checkpoint molecules such as programmed death-1, its ligand (PD-1/PD-L1), and cytotoxic T-lymphocyte-associated antigen 4 (CTLA-4). This section explores the immune cell components, signaling pathways, and tumor-associated biological processes that contribute to TNBC's net immunosuppressive state.

### Immune cell components of the TME in TNBC

The presence of tumor-infiltrating lymphocytes (TILs) in TNBC, such as CD8+ cytotoxic T cells, CD4+ helper T cells, and regulatory T cells, suggests a partial endogenous immune response to TAAs, which may present an opportunity to shift the balance toward antitumor immunity. TILs are comprised of a diverse group of immune cells that infiltrate tumors. TILs along with myeloid cells of the innate immune system, such as dendritic cells (DCs), tumor-associated macrophages (TAMs), and natural killer (NK) cells, are manipulated by the TNBC to form a pro-tumorigenic TME.

T cells play an important role in the TNBC immune state and dictate treatment response. The quantity, spatial distribution, and functional phenotype of TILs influence whether the TME is immune-activated or immunosuppressed.^9^ Compared to HR^+^ BC, TNBC exhibits higher levels of TILs, including increased intratumoral CD8^+^ T cells.^10^ Elevated TIL levels predict improved response to neoadjuvant chemotherapy, as well as enhanced disease-free and OS in early-stage TNBC, highlighting their unique role in TNBC compared to other BC subtypes.^11^ ^,^ ^12^ While the presence of TILs is important, their localization also affects clinical outcomes. CD8+ T cells correlate with survival benefits when distributed throughout the tumor rather than aggregating along the tumor border.^13-15^ Increased CD3^+^ and CD8^+^ infiltration has been correlated with increased frequency of pathologic complete response.^16^

Despite these findings, T-cell-mediated antitumor activity is often impaired in TNBC. Physical tumor-stromal barriers, including cancer-associated fibroblasts (CAFs) which are discussed later, can restrict T-cell infiltration. TNBC can also curb T-cell activity by inducing PD-1 expression, leading to T-cell anergy.^17^ Moreover, the composition of T-cell subsets can promote immunosuppression. TNBC tends to have more intratumoral CD4^+^ T cells and Tregs, and less intratumoral CD8^+^ T cells which biases the TME towards immune suppression.^18^ Some T-cell subsets can even contribute to tumor progression. For example, IL-17 released by Th17 cells enhances tumor growth by expanding MDSCs, despite also activating NK, B, and other T cells.^19^ Consistent with this picture of T-cell inhibition, recurrent TNBC tumors exhibit reduced immune activation and TILs compared to primary tumors, highlighting the importance of sustained T-cell activity for durable treatment efficacy.^20^

TNBC also acts on DCs to quell the antitumor immune response. Classical DCs (cDC) are primary antigen-presenting cells that consume TAA and cross-present them to T cells in lymph nodes, thereby initiating an adaptive immune response.^21^ Plasmacytoid DCs (pDC), a second subset of DCs, stimulate an immune response like cDCs, but differ in the mechanism. Typically, pDCs secrete type I interferons in response to viral or tumor nucleic acids, sustaining an immunogenic response.^22^ However, TNBC has more pDCs but less type I interferon compared to other BC types.^23^ Indeed, the TME can alter the maturation of pDCs by several mechanisms. For example, mouse BC-derived GM-CSF signals pDCs to initiate T helper 2 (TH2) immune responses that are generally anti-inflammatory and immunosuppressive.^24^ Furthermore, in the absence of a strong IFN-*α* response, tumor-associated pDCs bind to the inducible costimulatory molecule (ICOS) to promote proliferative expansion of immunosuppressive Tregs and portend a poor prognosis in TNBC.^23^

TAMs are a major component of the TNBC TME and play an important role in tumor progression. Although macrophages can suppress tumors, TNBC can induce their differentiation into subtypes that support tumor growth. Historically, macrophages were split into M1 and M2 subtypes. M1 macrophages, induced by Th1 cytokines, exhibit pro-inflammatory and tumoricidal activity, whereas M2 macrophages, driven by Th2 cytokines, reduce tumoricidal function.^25^ ^,^ ^26^ In TNBC, M2-like macrophages comprise most of the TAM population; however, this dichotomy oversimplifies macrophage biology in TNBC. Both M1 and M2 macrophages can coexist in the same TME and mixed phenotype M1/M2 macrophages have also been detected.^27^ Genomic and transcriptomic studies of TNBC tumors revealed TAM populations with overlapping M1- and M2-associated genomic signatures and cytokine profiles, implying a spectrum of functional states.^28^ ^,^ ^29^ Macrophage composition and polarization is clinically important. In TNBC, M1-like macrophages can induce tumor cell apoptosis and inhibit tumor invasion and metastasis.^30^ ^,^ ^31^ However, in TNBC, there is increased M1 to M2 polarization which promotes tumor progression and metastasis.^31^ TNBC samples with fewer M1-like macrophages demonstrated higher mutational burden, poorer ICI response, and shorter survival.^32^ Notably, even M1-like macrophages may exhibit atypical effects, such as chemotherapy resistance that promotes tumor growth.^33^ This highlights the complexity of TAM function in TNBC. Collectively, these findings highlight the need to reconsider the traditional M1/M2 framework and illustrate macrophages as complex, heterogenous immune cells that can contribute to TNBC progression. Therapeutic strategies should promote activation of TAMs towards pro-inflammatory, antitumor phenotypes.

NK cells can also demonstrate antitumor properties and TNBC secrete signals that alter and suppress NK cells, inhibiting direct tumoricidal immunity. Typically, higher NK cell infiltration is associated with improved response to ICI, but TNBC-associated NK cells can display altered function.^34^ A subpopulation of CD73-expressing NK cells was found to upregulate anti-inflammatory cytokines IL-10 and TGF-*β* via STAT3 transcriptional activity. These altered NK cells expressed high levels of PD-1, PD-L1, and LAG-3 to suppress T cells, leading to immune escape.^35^ This population of NK cells had an immature phenotype, indicating another layer of TNBC-driven immune cell reprogramming.

Thus, TNBC acts on major components of the innate and adaptive immune systems to suppress anticancer immunity.

### Immune pathways and checkpoints in TNBC

TNBC suppresses antitumor immunity at various points in the cancer-immunity cycle. TNBC cells have dysfunctional antigen presentation, which contributes to immune evasion. Low levels of MHC-I molecules, which are essential for internal TAA presentation and CD8+ T-cell activation, have been correlated with TNBC recurrence.^36^ Loss of MHC-I would consequently impair the effects of ICI or alternative T-cell-based therapy.^37^ Despite these derangements in antigen presentation, immunotherapy trials for TNBC do not focus on restoring this primary component of the cancer-immunity cycle.

Immune checkpoint signaling is another major mechanism for immune suppression in TNBC. Typically, PD-L1 is expressed on antigen-presenting cells (APCs), but BC and other solid tumors can increase the expression of PD-L1 on the surface of tumor cells, which binds to PD-1 on immune cells, primarily lymphocytes. Indeed, PD-L1 expression in ER/PR- BC has been associated with increased tumor size, grade, and proliferation.^38^ PD-L1 expression can differ between the immune system and tumors, as it is primarily expressed by immune cells (macrophages, CD4+ T-cells, and DCs) in some TNBC patients and by tumor cells in others.^39^ TNBC with higher PD-L1 expression had denser infiltration of CD4^+^/FOXP3^+^ Tregs in the stroma, suggesting that PD-L1 affects TME immune populations.^10^ PD-L1 also plays a direct role in tumor cell biology unrelated to its immunomodulatory functions. Nuclear PD-L1 is needed for chromatid formation, and depletion suppresses tumor growth in immune-deficient mice, indicating a function independent of immune evasion.^40^ Additional signaling pathways and epigenetic factors further mediate PD-L1 function.^41^ ^,^ ^42^ Unfortunately, PD-L1 expression alone is an unreliable predictor of response to ICI. Only 5–23% of TNBC cases preselected for high PD-L1 expression respond to monotherapy targeting PD-L1 or PD-1, with an inconsistent correlation between PD-L1 expression and ICI response.^43^ This limited predictive value reflects complex immune regulation in TNBC. Genetic analysis suggests that gene signatures incorporating multiple immune checkpoint pathways can predict immunotherapy response, highlighting the importance of checkpoints other than PD-L1.^44^ In TNBC, immune checkpoints LAG-3, TIM-3, and TIGIT can mediate immune evasion independent of PD-L1 in preclinical models.^45-47^ Heterogeneity in the TME and redundancy in immune checkpoint signaling likely contribute to the modest and inconsistent effects of ICIs in TNBC.

TMB is considered an indicator of immunogenicity and a predictor of responsiveness to ICI, but proves to be more complicated in TNBC. TMB is a measure of the overall level of somatic coding mutations occurring in a tumor specimen, reported as the number of somatic coding mutations per megabase (mut/Mb). TNBC tends to have higher TMB compared with other BC subtypes and *greater* tumor heterogeneity, but this is inversely associated with *fewer* TILs.^48^ ^,^ ^49^ A proposed etiology is the *immunoediting effect* during TNBC progression, in which TIL-rich TNBCs mount a robust antitumor immunosurveillance system which continuously and selectively eliminates highly mutative and immunogenic TIL clones, resulting in tumors with lower TMB. In fact, TNBC with higher TMB responds better to ICIs.^50^ Higher TMB in TNBC is associated with fewer TILs and a more immunosuppressive TME, suggesting greater potential benefit with immunotherapy. Conversely, low-TMB TNBC may exhibit limited neoantigen diversity, reducing ICI response and highlighting the need for alternative immune-stimulatory strategies in this subpopulation.

### Tumor biological processes altering the immune state

TNBC has unique structural characteristics that influence local immune functions and alter its response to therapy. The stroma is defined by an abundant population of fibroblast-like tumor stromal cells, called CAFs. These cells produce factors, such as vascular endothelial growth factor A (VEGFA) and TGF-*β*, that induce tumor progression by promoting angiogenesis, tumor growth and invasion.^51^ In TNBC, CAFs correlated with aggressive cancers, with features such as poor histological differentiation, recurrence, and lymph node metastasis that contribute to poor prognosis.^52^ CA9^+^ CAFs are a subset enriched in TNBC that interact with SPP1+TAMs, an immunosuppressive subset of macrophages, and lead to decreased T-cell infiltration, attenuate response to immunotherapy, and worsen prognosis.^53^ Current TNBC therapies do not target CAFs, which may contribute to the modest responses to therapy.

TNBC cells often undergo epithelial-to-mesenchymal transition (EMT), a normal embryologic or regenerative process that enables epithelial cells to become mesenchymal cells allowing migration for wound healing and tissue remodeling. TNBC cells progress from epithelial to mesenchymal during invasion and colony formation, with activation of multiple distinct EMT pathways during the metastatic cascade.^54^ More mesenchymal BC tumors contained more immunosuppressive cells (e.g. M2-like macrophages and Tregs) in the TME, limited CD8+ T-cell invasion, and exhibited increased resistance to anti-CTLA4 therapy.^55^ Even tumors with only ~10% mesenchymal tumor cells showed increased immunosuppressive Tregs and macrophages, spreading their protective effects to epithelial tumor cells.^55^ The EMT plays a central role in immune suppression and tumor progression. Disrupting EMT mechanisms could sensitize BC to immunotherapy, insinuating promise with new combination therapies.

### Limitations of immune checkpoint inhibition

As summarized in Table 1, derangements of signaling, cellular function, and tumor structure contribute to a net immunosuppressive TME in TNBC, which likely limits ICI efficacy. However, some patients do respond well to immunotherapy, implying that there is significant biological heterogeneity. While PD-L1 expression has been widely used for patient selection, emerging evidence suggest other better biomarkers. Maintenance durvalumab, a PD-L1 inhibitor, showed improved OS in TNBC compared to other breast cancer subtypes. However, neither PD-L1 status nor T-cell infiltration predicted response.^56^ This suggests that response to ICIs is dependent on tumor and microenvironment characteristics beyond PD-L1 status.

**Table 1.** Key mechanisms defining the immune microenvironment in TNBC.

Category	Component	Antitumor mechanism	Pro-tumor mechanism
Cellular components	Tumor-infiltrating lymphocyte (TIL)	CD8+ Cytotoxic T-cell invasion into the tumor leads to tumor killing and survival benefits	Anergic CD8+ T-cells accumulate at tumor border, high levels of immunosuppressive FOXP3+ Tregs
	Dendritic cell (DC)	Classical DCs cross-present tumor antigens to activate adaptive immunity; Plasmacytoid DCs secrete IFN-alpha to sustain immune response	Disturbed MHC-I/II impairs DC activation; TNBC-associated pDCs lose IFN-α secretion and promote Treg proliferation via ICOS
	Tumor-associated macrophage (TAM)	M1 macrophage induce tumor cell apoptosis and inhibit invasion	TNBC induces anti-inflammatory M2 macrophage polarization, promoting tumor growth and metastasis
	Natural killer (NK) cell	High intratumoral NK cells kill tumor cells and sensitize TNBC to chemotherapy	TNBC induces immature NK cells which upregulate IL-10/TGF- β and Wnt ligands to suppress T-cell function and promote tumor growth
Immune signaling	Antigen presentation	MHC-I presentation of tumor-associated antigens (TAA) activates antitumor CD8+ T-cells and reduces tumor recurrence	TNBC downregulates MHC-I which impairs T-cell activation and attenuates ICI response
	Checkpoint molecules	High PD-L1 expression sensitizes TNBC to checkpoint inhibitors	PD-1/PD-L1 signaling inactivates T-cells; response to checkpoint inhibitors is inconsistent in PD-L1+ TNBC
	Tumor mutational burden (TMB)	Cancers with elevated TMB can produce more neoantigens and enable greater immune infiltration	Immunoediting and similar immunosuppressive mechanisms increase TMB and TNBC tumor heterogeneity with fewer TILs
Biological processes	Cancer-associated fibroblast (CAF)	N/A	CAFs are found in aggressive TNBC and promote angiogenesis, tumor invasion, and immunosuppression
	Epithelial-to-mesenchymal transition (EMT)	N/A	TNBC undergoes EMT, driven by TGF- β signaling, which leads to immune suppression, metastasis, and resistance to immunotherapy

TNBC = triple-negative breast cancer, ICI = immune checkpoint inhibitor.

Multiple studies further demonstrate limitations of PD-L1 as a predictive biomarker and identify alternate markers. PD-L1 expression does not consistently correlate with treatment response, whereas intact MHC-I predicted complete treatment response.^57^ This highlights the importance of antigen presentation. In addition to PD-L1, TME gene signature and DNA damage patterns predict response to ICI with chemotherapy in TNBC.^58^ Alternate immune markers might be better predictors of immunotherapy response than PD-L1 expression.

TNBC can be classified into subtypes by genomic and transcriptomic signatures with differential response to immunotherapy. Low hypoxia gene and high immune cytokine expression correlate with OS in TNBC patients.^59^ These gene signatures may reflect differences in tumor immunomodulation. A large study separated 465 patients with refractory metastatic TNBC into transcriptome-based subtypes, including luminal, immunomodulatory, immune-suppressed, and mesenchymal-like.^60^ Based on these subtypes, patients were then treated with one of seven targeted combination therapies resulting in improved response rates.^61^ As mentioned earlier, TNBC’s TME is heterogenous and specific immune cell populations can change outcomes. For example, a subtype enriched in CXCL9+ macrophages had improved ICI response, and addition of an IDO1 inhibitor further enhanced efficacy.^62^ There are many pathways and variant immune signaling beyond PD-L1 status that differentiate TNBC patients and affect response to immunotherapy. One study reported 28% of 386 TNBC patients possessed a “hot” TME, characterized by increased immune cell infiltration and better relapse-free survival.^63^ These findings suggest that limited ICI efficacy in TNBC is due to immune evasion, an immunosuppressive TME, and inadequate biomarker-based patient selection.

### Implications for treatment

Given these limitations, strategies that actively remodel the TNBC TME are required to enhance immunotherapy outcomes. These mechanisms contribute to patient heterogeneity, recurrence risk, prognosis, and therapeutic efficacy.

One way to circumvent this challenge is to shift the TME towards immune activation, thereby enhancing the immune system's antitumor response. Combination approaches further improve outcomes. Anti-angiogenic agents such as apatanib and cytotoxic agents like eribulin induce vascular remodeling and immune infiltration, improving immunotherapy outcomes.^64^ Similarly, inhibition of tumorigenic pathways, such as STAT1 and CXCR4, in mice models reduces MDSCs and T-cell exhaustion, enhancing response to PD-L1 inhibition.^64^ ^,^ ^65^ Patient selection based on immune populations also affects efficacy. TNBC enriched in antigen-presenting mast cells within tertiary lymphoid structures responded better to PD-1 inhibitors, which can be further enhanced by cromolyn.^66^ In tumors with more CD8^+^ T cells, combined antiangiogenic therapy with PD-1 inhibition yields high response rates, particularly in PD-L1^+^ patients.^67^ While PD-L1 status can suggest treatment efficacy, durable treatment responses require better patient selection and addition of immune adjuvants.

Immune activation and an antitumor response relies on antigens generated by the tumor. As such, new therapeutic strategies that enhance antigen release to activate the TME could be beneficial, and cryoablation represents one such approach. The next sections will focus on cryoablation as a source of TAA and its potential to synergize with immunostimulants to enhance responses to immunotherapy.

## Current and emerging roles of cryoablation for breast cancer

Cryoablation is a minimally invasive, nonsurgical technique that uses extreme cold to locally destroy tumors, including BC. This section reviews the current role of cryoablation and explores its potential as an adjunct immunotherapy.

### Current role of cryoablation in breast cancer

Cryoablation of breast tumors is becoming more common as an alternative to surgery in select patients. The superficial location of BC within breast tissue and its conspicuity on ultrasound imaging facilitates the percutaneous placement of the cryoprobe. Cryoablation is also well-tolerated by patients due to the anesthetic effect of ice, and the dermotomy requires no sutures. Recovery is faster than surgery, with patients returning to normal activities within days.^68^

Multiple studies report the safety, feasibility, and efficacy of cryoablation in achieving local control of small, early-stage BC tumors.^69-71^ The ongoing ICE3 and FROST trials are measuring long-term recurrence when using cryoablation instead of surgery in BC patients. Interim data in the ICE3 trial indicates a similar rate of regional recurrence in the cryoablation subgroup with lower risks than surgery.^71^ Although these studies use cryoablation to achieve complete tumor ablation, subtotal or incomplete cryoablation could be used to generate TAA as part of an in situ cancer vaccination approach against TNBC.

**Figure 1.**
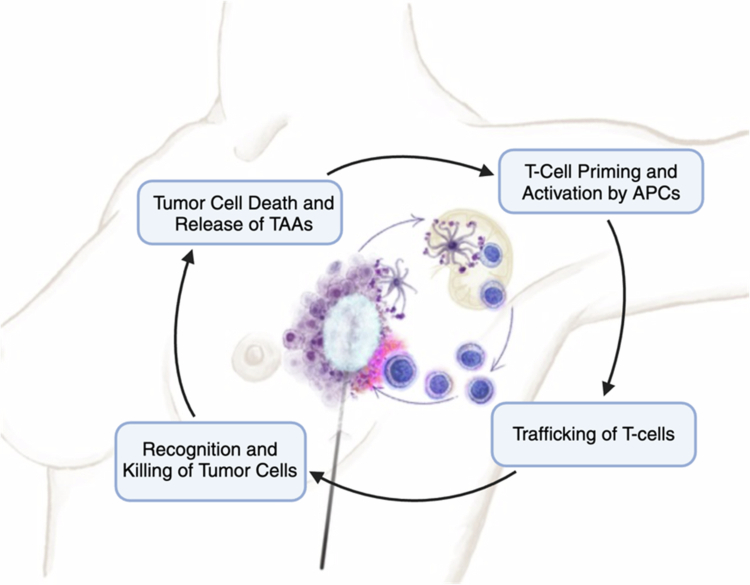
Cancer immunity cycle in TNBC. Cryoablation (depicted by cryoprobe) induces cell death and release of tumor-associated antigens. These antigens are consumed and presented by APCs at local lymph nodes, where T-cells are primed, activated, and cloned to target TAAs. These T cells are then trafficked towards the tumor site, where tumor cells are selectively bound and destroyed by effector T-cell mechanisms, producing more TAAs and resupplying the cycle.

Cryoablation has garnered attention as an immune-stimulatory modality because freeze‒thaw injury yields abundant TAAs and stimulates a robust inflammatory response (Figure 1). Unlike thermal ablation, the TAAs generated by cryoablation are intact and not denatured. Cryoablation creates an ice ball that can reach temperatures as low as −190 °C, producing a central zone of direct injury where intracellular ice formation destroys the plasma membrane. Repeated freeze‒thaw cycles further create osmotic changes, causing cell shrinkage followed by swelling and membrane rupture.^72^ Cells not immediately destroyed undergo two predominant cell death mechanisms—necrosis and apoptosis—and the type of immune response depends on the mechanism. Temperatures below −20 °C induce necrosis, a passive cell death modality characterized by loss of membrane integrity and rapid release of cytoplasmic and nuclear contents.^73^ This release of TAAs induces inflammation, thereby facilitating immune recognition.^74^ In contrast, apoptosis occurs at milder subfreezing temperatures, typically at the periphery of the ice ball.^75^ ^,^ ^76^ Apoptotic cells do not rupture and are rapidly cleared by phagocytes, making apoptosis immunologically silent.^73^ Cryoablation induces apoptosis through caspase-mediated mechanisms: an extrinsic membrane pathway at ultra-low temperatures or an intrinsic mitochondrial pathway at warmer freezing temperatures.^74^ ^,^ ^77^ ^,^ ^78^ However, the extrinsic pathway is limited to the first few hours after cryoablation, as cells transition to necrosis within 24 hours, making the intrinsic pathway the primary driver of long-term immune responses. Both necrosis and apoptosis contribute to tumor cell death. However, these cell death mechanisms differ by their immune effects.

### Cryoablation for stimulating an anti-BC immune response: opportunities

The immunostimulatory effects of cryoablation are multifactorial. In preclinical and clinical studies, cryoablation has been noted to be immunogenic in some settings and immunosuppressive in others.^72^ The necrotic core of the ablation zone is a reservoir for intact TAAs and damage-associated molecular patterns (DAMPs), including heat shock proteins (HSPs).^72^ Cryoablation induces inflammation and hyperemia, ushering immune cells to the ablation zone periphery. It elevates pro-inflammatory factors IL-1, IL-6, TNF-*α* and NFκB in liver and lung cancer that exceed those induced by heat-based thermal ablative modalities.^79-82^ IL-6 has been described both pro- and antitumorigenic, and its role differs between acute postcryoablation inflammation and the chronic inflammation induced by BC. Cryoablation also suppresses Tregs by reducing TGF- β in mouse adenocarcinoma.^83^ Immunostimulants may further shift cytokines toward antitumor behavior. Nondenatured TAAs released by cryoablation are taken up, processed, and presented by APCs (e.g., DCs or macrophages) on MHC molecules for T-cell priming. DAMPs are phagocytosed by DCs and activate the NF-κβ pathway, promoting costimulatory molecule expression.^82^ The combination of APC TAA presentation and expression of costimulatory molecules activates T cells and promotes a systemic immune response.^77^ ^,^ ^84^ These preclinical studies establish a robust mechanistic basis for the cryoablation's immunogenic potential.

Clinical studies in non-TNBC cancers support these immune effects. The addition of cryoablation to PD-1 inhibition (vs chemotherapy + PD-1 inhibition) increased the CD4^+^/CD8^+^ ratio and improved survival, response rates, and disease control in melanoma and lung cancer.^85^ ^,^ ^86^ Cryoablation increases tumor immunogenicity, DC activation, and intratumoral CD8^+^PD-1^+^ T cells in cholangiocarcinoma.^87^ In non-small-cell lung cancer, cryoablation improves survival relative to thermal ablation and increases IFN- α .^88^ In RCC, cryoablation alone expands multiple immune cells populations and Th1 cytokines.^89^ Together, these data suggest an immune-activating role of cryoablation, though translation to TNBC is limited due to a lack of BC-specific studies.

Rarely, cryoablation can trigger a phenomenon termed the “abscopal effect”: a systemic antitumor immune response that can eliminate distant metastases and generate immune memory.^90^ ^,^ ^91^ Animal studies support this phenomenon. In a BC rechallenge model, only 16% of mice previously treated with cryoablation developed tumors, compared with 86% after surgery.^74^ ^,^ ^91^ However, clinical translation is limited. Only isolated case reports have described an abscopal response in BC, with minimal evidence in TNBC.^92^ ^,^ ^93^ Although inducing a reliable abscopal effect remains challenging, cryoablation may represent an *in situ* tumor vaccine strategy for TNBC. Given TNBC’s immunogenic features but overall immunosuppressive TME, combination approaches are likely required to consistently induce an abscopal effect.

Cryoablation technique and procedural variables can alter its immune effects. High freeze rates increase tumor-specific T cells, reduce pulmonary metastases, and improve survival, while low freeze rates increase Tregs and worsen survival relative to surgery.^91^ Increasing freeze-thaw cycles or number of probes is associated with larger regions of necrosis, likely releasing more TAAs and enhancing immune activation in vitro.^94^ In vitro prostate cancer models show that extended freeze cycle length, more cycles, and passive thawing maximize cell destruction and likely maximize TAA release.^95^ These experimental studies suggest technical cryoablation methods that could further improve its immune activation, although this data requires validation in vivo and in controlled clinical studies.

Direct comparisons with thermal ablation are limited and inconsistent. In a mouse colon cancer model, cryoablation and RFA both induced systemic antitumor effects, with RFA showing fewer Tregs in the ablation zone and cryoablation eliciting a more diverse inflammatory cytokine response.^96^ In colon cancer patients, however, cryoablation caused greater release of tumor gangliosides and IgM against these gangliosides compared to RFA, consistent with necrosis-mediated release of preserved TAAs.^97^ In renal cancer patients treated with cryoablation, MWA, or RFA, cryoablation produced the largest change in serum inflammatory cytokines and proteins, which increased with cryoprobe number.^98^ Although multiple clinical and preclinical studies have validated the immunomodulatory effects of cryoablation, sample sizes are low and correlation between ablation modality, immune function, and clinical outcomes are limited. Overall, both cryoablation and heat-based ablation are immunogenic.

### Cryoablation for stimulating an anti-BC immune response: challenges

Wide variations in immune responses following cryoablation may reflect technical differences in the cryoablation procedure. These variations include the freeze‒thaw rate, lowest temperature reached, duration at lowest temperature, number of freeze‒thaw cycles, and size of the lesion treated, discussed earlier.^93^ Currently, there is no set of cryoablation parameters that can consistently produce desired immunogenic effects, which is a current barrier to further adoption of the technique.

Along the periphery of the cryoablation ablation zone, sublethal hypothermia causes mitochondrial injury, leading to apoptosis. Apoptotic cells can release a limited quantity of TAA, but few DAMPs (HSP, DNA, RNA, HMGB1) are released. APCs face an immunosuppressive environment when they encounter an apoptotic tumor cell due to a lack of costimulatory signaling and release of immunosuppressive cytokines such as IL-10 and TGF-*β* by macrophages.^99^ Lack of APC activation with immunosuppressive cytokines leads to T cell anergy or clonal deletion with increased Tregs that effectively produces a quiescent apoptotic TME.^90^ These effects would be expected to compound the immunosuppression in the TNBC TME. These limitations are reflected in clinical studies. In one small BC study, cryoablation alone did not activate effector CD4^+^ or CD8^+^ T cells, or increase Th1 cytokines.^100^ However, cryoablation increased T cells and Th1 cytokines in HER2^+^ BC.^101^ Immunotherapy combined with cryoablation has been tested as a way to overcome an immunosuppressive TME.

Combination therapies with cryoablation, however, require caution due to the potential for immune over-activation. Cryoablation's immunogenicity can produce severe systemic inflammation. Large-volume liver ablation has been associated with “cryoshock”, an inflammatory phenomena characterized by thrombocytopenia, disseminated intravascular coagulation (DIC), renal and hepatic failure, and ARDS.^102^ ^,^ ^103^ Historically reported in ~1% of cases, cryoshock now occurs in only ~0.265% of cases, with major complications below 5%, due to the change from open to percutaneous ablation, smaller targets, improved freezing gases, and better imaging and temperature monitoring.^104^ In contrast to the expected abscopal effect, one study in hepatoma showed that IRE-induced inflammation accelerated secondary tumor growth, which was mitigated with administration of an anti-IL-6 antibody.^105^ Although uncommon, these events remain important when weighing procedural risk, particularly when combined with immunotherapy. Reassuringly, clinical studies combining cryoablation with ICIs report good overall tolerability, with mostly grade 1–2 adverse events (AEs) and no significant increase compared to monotherapy.^88^ ^,^ ^106^ Immune-mediated toxicities from cryoablation may be amplified by additional immune-activating agents.

### Clinical trials of cryoablation plus immunotherapy for TNBC

There are few trials combining immunotherapy with cryoablation, especially in TNBC. In a small trial of 19 BC patients, cryoablation combined with ipilimumab increased Th1 cytokines, expanded activated CD4^+^ and CD8^+^ T cells, and reduced the proportion of Tregs, while providing a favorable safety profile (only one rash reported).^100^ A second study using the same regimen in early-stage BC increased TIL clonality.^107^ This suggests rapid T-cell expansion from increased TAA release after cryoablation. Ongoing clinical trials of immunotherapy with cryoablation for TNBC focus on its combination with ICI. A Phase 1 trial is evaluating whether cryoablation can improve the response of metastatic or locally advanced TNBC to pembrolizumab monotherapy.^108^ A similar phase 1/2 trial is evaluating single dose pembrolizumab monotherapy combined with cryoablation for high-risk TNBC compared to cryoablation alone or surgical excision alone.^109^

A phase 2 trial is studying ICI (pembrolizumab monotherapy as compared to dual ICI therapy with CTLA-4 inhibitor ipilimumab and PD-1 inhibitor nivolumab) administration both before cryoablation and after surgical excision.^110^ These trials are based on the hypothesis that cryoablation will promote antigen presentation by providing abundant TAA and the ICI will disinhibit T cells to permit antitumor immune recognition. Whether this combination will be sufficient to overcome the immunosuppressive TME of TNBC remains to be determined. It is likely that additional co-therapies will be required to tip the balance of the TME towards an antitumor immune response. Section 4 will extend the discussion to include immunoadjuvants, which increase the immunogenicity of the TME.

## Cryoablation combination therapies in breast cancer

**Figure 2.**
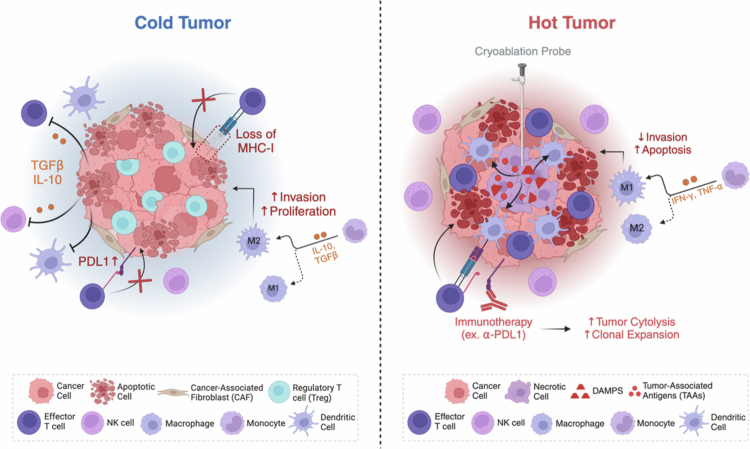
Immunologically hot and cold tumor with TME-specific immune mechanisms in TNBC. The cold tumor is dominated by an immunosuppressive TME with anti-inflammatory cytokines (IL-10, TGF-*β*) released by tumor cells and CAFs. Downregulation of MHC-I by tumor inhibits antitumor immune populations (DCs, NK cells, effector T cells) and induce immunosuppressive Tregs and M2 macrophages. Cryo with immunotherapy can generate a hot tumor that increases infiltration and activation of antitumor effector T cells, DCs, and NK cells. This TME is dominated by pro-inflammatory cytokines TNF-*α* and IFN-*γ* that stimulate immune cell expansion and cytotoxic activity, leading to reduced tumor cell invasion and increased death.

If these studies of TNBC show that adding cryoablation to ICI is insufficient to reliably provoke an effective antitumor immune response, an immunostimulant may be indicated to shift the balance towards immune recognition and elimination of tumor cells. While ICI removes the brakes on CD8+ T cells, immunostimulants act as immunologic flairs that direct the immune system to the site of TAA, thereby augmenting the immune response to nearby antigens and enhancing APC antigen presentation.^111^ ^,^ ^112^ For TNBC, the addition of immunostimulants to cryoablation and ICI could convert these immunologically cold or warm tumors into hot ones, seen in Figure 2.

### Opportunities for immunostimulants with cryoablation

Immunostimulants act through a variety of immune mechanisms to exert different effects on the TME, which could amplify the effects of cryoablation in TNBC. CpG oligodeoxynucleotides (ODNs) are synthetic immunostimulants that bind to Toll-like receptor 9 (TLR9) within endosomal compartments of B cells and pDCs to trigger the upregulation of IFN-*α*, IFN-ß, and proinflammatory cytokines such as TNF-*α*, IL-6, and IL-12.^113^ The combination of CpG ODNs with synthetic antigens induced TAA-specific cytotoxic T cells, and specifically inhibited growth of TNBC in in vivo.^114-116^ Cryoablation plus CpG increased tumor infiltration of CD8+ T cells, curbed tumor growth, and extended survival time points in a murine model of liver cancer, which also has an immunosuppressive TME.^82^ ^,^ ^115^ CpG ODN with cryoablation enabled antigen-CpG colocalization in DCs, thereby amplifying immunotherapy effect.^116^ Intratumoral CpG ODN have been tested in patients across multiple cancer types, including melanoma, lung cancer, glioblastoma, and head and neck squamous cell carcinoma, demonstrating excellent tolerability but only modest-to-no clinical efficacy.^117-120^ In TNBC, intratumoral CpG ODN combined with cryoablation could stimulate pDCs and counteract the immunosuppressive cryoablation periphery, where limited DAMP release promotes T cell anergy or clonal deletion.

Although not yet tested in TNBC, a phase II trial of CpG ODN with trastuzumab in metastatic HER2^+^ BC showed fluctuations in immune cell and cytokine levels without clear immunological trends except for increased MDSCs, while the combination demonstrated increased OS.^121^ CpG ODN likely produces immune activity that contributes to tumor response, though effects may be modest or not fully captured with available assays used. CpG has shown stronger activity in metastatic melanoma, increasing CD8^+^ T cells, NK cells, DCs, and B cells, producing IL-12 and type I interferon, achieving overall response rates up to 80% in both PD-1^+^ and PD-1^-^ tumors, and producing a 15% response rate in PD-1-resistant disease.^122^ ^,^ ^123^ This suggests potent immune activation, even in tumors unresponsive to ICI. However, efficacy of CpG can vary. In metastatic RCC, patients with elevated baseline IFN- γ had better CpG vaccination response, marked by increased activation and reduced number of pDCs.^124^ This suggests CpG could benefit from combination with a second immunogenic therapy. Delivery improvements, such as biomaterial scaffolds, can further enhance CpG ODN spatial release and immune activation.^125^ CpG ODN shows promise in improving clinical outcomes, although delivery and combination therapies need to be optimized and tested in TNBC.

**Figure 3.**
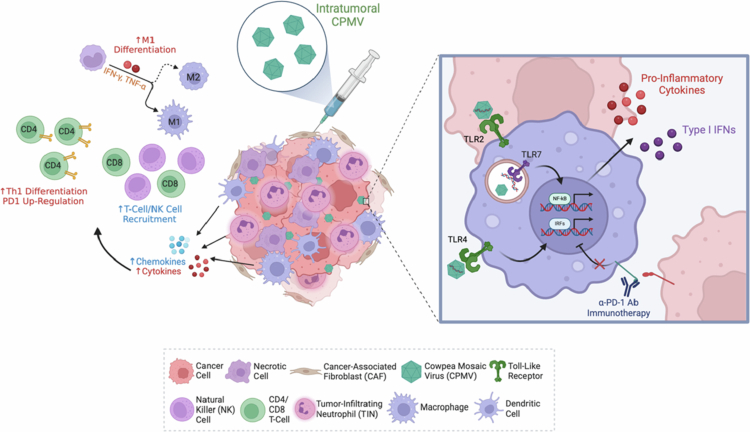
Intratumoral CPMV and antitumor effects on TME of TNBC. CPMV particles initially bind and activate TLR receptors on phagocytic cells, including tumor-infiltrating neutrophils (TINs), macrophages, and dendritic cells (DCs). The CPMV capsid activates TLR2/4 on the cell membrane while its contained mRNA activates endosomal TLR7. This leads to NF-kB and IRF gene expression that produces pro-inflammatory cytokines and type I interferons. Inflammatory cytokine/chemokine signaling recruits more immune cells and activates them towards an antitumoral phenotype.

Another promising immunostimulant is Cowpea mosaic virus (CPMV) with preclinical utility for the treatment of canine cancer patients as an in situ vaccine for a variety of cancers, including BC. CPMV as well as virus-like nanoparticles (VLPs), devoid of the viral genome, have been studied in tumor mouse models and canine cancer patients.^126^ ^,^ ^127^ CPMV promotes an antitumor response via TLR2, 4 and 7 stimulation.^128^ CPMV has been shown to promote the expression of several immune checkpoint molecules on CD4+ T cells in different tumor models and potentiates the benefit of ICI.^129^ ^,^ ^130^ Co-culturing CPMV with macrophages or bone marrow derived DCs resulted in increased levels of pro-inflammatory cytokines.^127^ These studies indicate that in situ CPMV therapy remodels the immunosuppressive TME into a pro-inflammatory one which is summarized in Figure 3.

Preclinical data has demonstrated the efficacy of CPMV against BC in animal models. The combination of CPMV and cyclophosphamide in a mouse TNBC model demonstrated a synergistic effect by inhibiting the growth of primary tumors and suppressing a metastatic lung tumor, suggesting an abscopal effect.^131^ In canine mammary cancer patients, intratumoral CPMV led to tumor shrinkage in 10 of 11 dogs and tumor reduction in most of the untreated (noninjected) tumors, both in the ipsilateral and contralateral mammary tumors. Increased neutrophil and lymphocyte invasion and DC activation was also observed in the TME, suggesting induction of the abscopal effect.^132^ In a murine model of liver cancer, an abscopal effect was only observed when intratumoral CPMV was combined with cryoablation.^133^ Thus, combining CPMV with cryoablation may produce a durable anti-TNBC immune effect that would reduce recurrence and improve survival. However, CPMV has not been tested in humans, so it currently is a promising strategy that requires significant clinical translation before it can be introduced into TNBC treatment.

Several other immunostimulants have been tested directly in TNBC. Meriva, a plant-derives polyphenol, reduces IL-6 through NF-kB and has anticancer activity.^134^ In a TNBC mouse mode, Meriva combined with cryoablation activated CD8^+^ T cells against TAAs, significantly improved survival, and reduced metastatic growth.^135^ The combination outperformed monotherapy, indicating a synergistic effect. Adoptive cell therapy has also shown promise. In HER2^+^ BC, cryoablation alone and combined with NK cell therapy increased T cells and Th1 cytokines. The combination also increased NK and B cells and produced nonsignificant reductions in tumor size, with median survival increase from 9 months to 12 months.^101^ Although limited, these findings suggest enhanced immune function with cryoablation-based combinations.

## Limitations of immune stimulants

Despite their potential synergistic role with cryoablation, systemic immunostimulants carry safety considerations. Immunostimulants used intratumorally, however, are generally well tolerated, with low rates of serious AEs (2–4%) rate of serious AEs, and predominantly mild, localized reactions such as injection-site pain or inflammation.^136^ ^,^ ^137^ Rare cases of cytokine release syndrome, a systemic inflammatory response, have been reported and can be severe, though they are uncommon and manageable.^136^ ^,^ ^138^ In contrast, systemic immunotherapy carries substantially higher toxicity. Ipilimumab in melanoma produced 20–36% grade 3–4 AEs, inducing diarrhea and colitis, with up to 74% of patients experiencing an immune-related AE at high dose.^139^ Locoregional delivery has been shown to mitigate this risk.^140^ However, not all systemic immunotherapy is toxic, such as a chemokine regimen that was very well tolerated in TNBC.^141^

Combination therapies demonstrate variable safety profiles. Intratumoral agents used alone or with PD-1 inhibitors show manageable toxicity without clear increases in immune-related side effects. In TNBC, talimogene laherparepvec (T-VEC), an oncolytic virus, combined with PD-L1 blockade had favorable tolerability with few serious AEs.^142^ ^,^ ^143^ However, IL-12 gene therapy combined with chemotherapy and PD-1 inhibition has shown high toxicity, including 90% of patients with grade ≥ 3 AEs and two treatment-related deaths.^144^ Overall, while immunotherapy carried important risks, intratumoral delivery offers a more safety profile. Careful selection and evaluation of combination regimens is essential.

Viral-based nanoparticles face significant regulatory hurdles. FDA guidelines for characterization, toxicity, and environmental impact are still evolving.^145^ Nanomaterials are diverse, so standardization and regulatory frameworks for development of particles is needed.^146^ ^,^ ^147^ Plant-based particles raise concern for allergic responses, though early clinical trials of virus-like particles have demonstrated safety.^148^ ^,^ ^149^ Significant steps need to be taken before viral nanoparticles are implemented into clinical practice.

Finally, TNBC has a significant amount of genomic and TME heterogeneity which affects its response to immunotherapy, as discussed earlier. TLR expression varies TNBC subtypes and between patients, suggesting differential responsiveness to TLR-based immunotherapies like CpG-ODN.^150^

## Conclusion

Novel strategies to incite an antitumor immune response against TNBC could improve ICI treatment response and survival. Cryoablation holds promise for the treatment of TNBC but is insufficient for reliably provoking a systemic antitumoral immune response instead of immune tolerance. Combining cryoablation, a source of abundant TAA, with immunostimulants and ICI could tip the balance towards immune activation by provoking elimination of tumor both locally and at distant sites via the abscopal effect. Tailoring Cryo-immunotherapy regimens based on the TNBC immune signature will be crucial for achieving improved patient outcomes. Preclinical studies are needed to define which combinations of immunostimulants, ICI, and cryoablation would be safest and most effective for different TNBC TMEs. If successful, clinical trials of partial cryoablation plus intratumoral immunostimulants and ICI could be initiated for patients with inoperable TNBC or metastatic disease.

## Data Availability

No datasets were generated or analyzed in this study.
